# Mitochondria-Targeted Lonidamine Hydrogels for Postsurgical Elimination of Glioblastoma

**DOI:** 10.3390/gels12080682

**Published:** 2026-08-03

**Authors:** Li Fan, Yuhan Sun, Guangzhao Lu, Lijia Kong, Yangyuyuan Song, Rongrong Yu, He Zhang, Wei Wu, Huan Wang, Ying Lu

**Affiliations:** 1 Department of Pharmaceutical Sciences, School of Pharmacy, Naval Medical University, Shanghai 200433, China; fanli7062022@163.com (L.F.); sunyuhan0101@163.com (Y.S.); lu5510647@163.com (G.L.); 13665530952@163.com (L.K.); syyy618@163.com (Y.S.); yurr1767@163.com (R.Y.); wwzh22@126.com (H.Z.); 2 Key Laboratory of Smart Drug Delivery, Ministry of Education, School of Pharmacy, Fudan University, Shanghai 201203, China

**Keywords:** peptide drug conjugates, lonidamine, glioblastoma, mitochondria, supramolecular hydrogel

## Abstract

The high recurrence rate of glioblastoma after surgical resection and conventional chemotherapy remains a major obstacle to effective treatment. Mitochondria-targeted long-acting local chemotherapy represents a promising therapeutic strategy to tackle this dilemma. In this study, a mitochondria-penetrating peptide (mito) was conjugated with lonidamine (LND), a mitochondrial hexokinase II inhibitor, to construct an amphiphilic peptide–drug conjugate (LND-mito) that could self-assemble into supramolecular hydrogels. This design achieves sustained localized release and mitochondria-targeted delivery, generating a tumor-selective oxidative phosphorylation inhibitor with 19-fold higher potency than LND. Our results demonstrated that LND-mito efficiently targeted tumor cell mitochondria, induced robust reactive oxygen species generation, decreased mitochondrial membrane potential, and activated mitochondrial apoptosis, ultimately markedly inhibiting glioma growth and prolonging postoperative survival in orthotopic glioma-bearing mice. Collectively, this study offers a promising therapeutic strategy for glioma management, while simultaneously informing the design of high-efficacy, low-toxicity local sustained-release delivery platforms for malignant tumors.

## 1. Introduction

Glioblastoma stands out as the most diffusely invasive and aggressive primary brain tumor. Despite standard frontline therapies, complete tumor eradication remains unattainable, and recurrence is inevitable. The median overall survival in clinical trials ranges from 14.6 to 16.7 months, with the 2-year survival rate being barely 26–33% [1,2,3,4]. Currently, the standard management of glioblastoma involves maximal surgical resection, followed by postoperative radiotherapy and adjuvant chemotherapy initiated approximately two weeks after surgery. This strategy may miss the optimal therapeutic window for eradicating residual tumor cells, while chemotherapy efficacy is further severely limited by the blood brain barrier [5,6,7]. In contrast, implanting a sustained-release drug delivery system directly at the resection cavity immediately after surgery allows high-concentration drugs to cover the tumor bed, effectively eradicating residual tumor cells, minimizing systemic toxicity, and bridging the therapeutic gap between surgery and systemic treatment. For instance, the GLIADEL^®^WAFER (carmustine implant) has been clinically validated to enhance patients’ quality of life and prolong progression-free survival. However, despite its clinical efficacy, it is limited by short release duration, limited tissue penetration, and failure to achieve sustained, stable local chemotherapy [8,9].

Injectable hydrogels can mimic tissue physiological environments, making them ideal sustained-release carriers for local drug delivery [10,11,12,13]. Conventional polymeric injectable hydrogels physically loaded with therapeutic agents have been widely applied for local tumor combination treatment [14,15,16], yet they are limited by burst release, foreign-body inflammation and absence of intracellular targeting. Compared with traditional hydrogels, natural amino acid-synthesized peptide–drug conjugate (PDC) hydrogels have advantages of precise self-assembly, low immunogenicity, and easy modification. Rational peptide sequence design enables multifunctional structures and incorporation of functional targeting peptides further allows drug self-delivery [17,18,19,20]. Leveraging these advantages, amphiphilic PDCs can form supramolecular hydrogels under appropriate conditions, thereby enabling local sustained drug release [21,22]. Notably, targeted modified amphiphilic PDCs combine hydrogel sustained-release properties with modified drug targeting, achieving a synergistic therapeutic effect.

As a major ATP producer and apoptosis regulator, mitochondria represent a promising target for suppressing tumor proliferation and metastasis by disrupting tumor bioenergetics [23,24,25]. Lonidamine (LND), an anti-glycolytic agent, acts on mitochondrial hexokinase II to suppress aerobic glycolysis and trigger apoptosis in cancer cells [26,27,28]. Therefore, the development of mitochondria-targeted LND could significantly enhance its antitumor potency by concentrating its activity within mitochondria. Mitochondrion-penetrating peptides (mitos) are short peptides capable of crossing both cell and mitochondrial membranes, allowing them to serve as effective mitochondrial targeting moieties to improve the intracellular and mitochondrial delivery of LND. Furthermore, the hydrophilic amino acid region of mitos acts as the hydrophilic domain of amphiphilic molecules, enabling the LND-mito conjugate to self assemble into a hydrogel [29,30,31].

**Figure 1:**
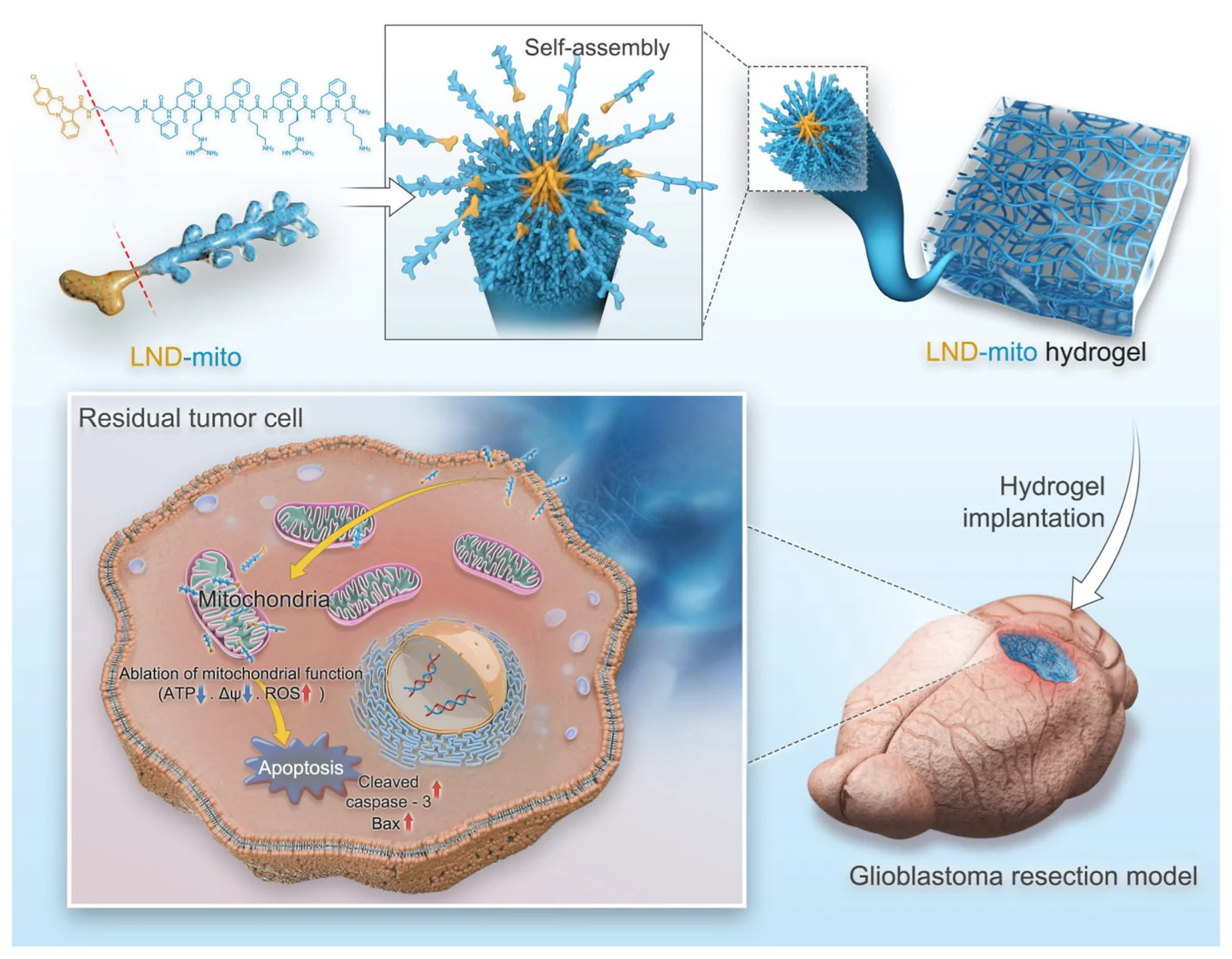
Schematic illustrations of the LND-mito hydrogels drug delivery system.

In this study, we designed a mitochondria-targeted hydrogel for sustained LND-mito delivery (Figure 1). This platform implemented a drug self-delivery strategy in which the amphiphilic LND-mito conjugate acted as both the therapeutic agent and the hydrogel scaffold, eliminating the need for additional carriers. The LND-mito hydrogels displayed favorable gelation behavior and controlled drug release profiles, enabling efficient mitochondrial targeting, ROS elevation, mitochondrial depolarization, and ATP depletion. These effects induced apoptosis in GL261 cells and prolonged survival in mouse models of subcutaneous and orthotopic glioblastoma after surgical resection.

## 2. Results

### 2.1. Design and Characterization of LND-Mito Hydrogels

The mito sequence was rationally designed to possess two key features essential for efficient translocation across both cellular and mitochondrial membranes: positive charge and lipophilicity. In general, a mito consists of alternating hydrophobic (e.g., phe) and cationic (e.g., lys) residues, which endow them with superior cell permeability and selective mitochondrial targeting ability. Based on this design principle, we synthesized a lonidamine conjugated peptide (LND-mito, LND (CH_2_)_5_ CONH FFDRFKFDRFK NH_2_) by coupling LND to the mito via an aliphatic linker (Figure S1) [32,33]. The successful synthesis of LND-mito was verified by mass spectrometry and high-performance liquid chromatography (Figure S2).

**Figure 2:**
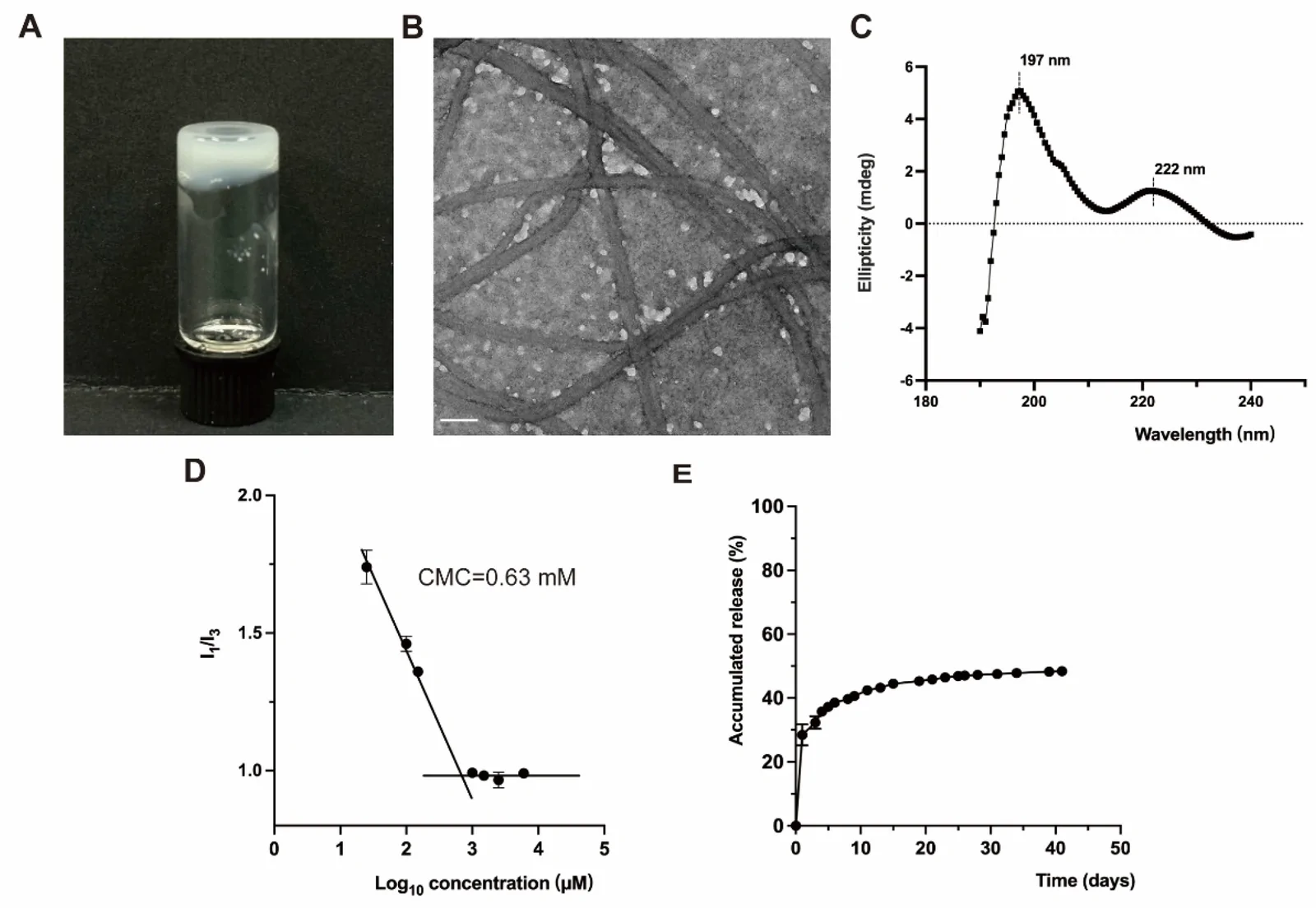
Characterization of LND-mito hydrogels. (**A**) Hydrogelation behavior of LND-mito. (**B**) Typical TEM micrograph of the LND-mito hydrogel. Scale bar = 200 nm. (**C**) Representative CD spectrum of LND-mito in aqueous solution. (**D**) Critical micelle concentration of LND-mito. (**E**) Cumulative release profiles of LND-mito from the hydrogel. Data are means ± SDs (*n* = 3).

Given its amphiphilic nature, LND-mito can spontaneously self-assemble into supramolecular hydrogels in aqueous environments (Figure 2A). Such self-assembly behavior is favored by the good water solubility of LND-mito. Using a vial inversion method, the critical gel concentration of LND-mito in PBS was determined to be 140–145 mM. After aging for 6 h at room temperature, self-assembled LND-mito morphology was examined by transmission electron microscopy (TEM). Figure 2B showed a uniform nanofibrous network with an average diameter of approximately 32 nm formed by LND-mito. Circular dichroism (CD) spectroscopy further demonstrated that the secondary structure of LND-mito was predominantly composed of β sheets (Figure 2C). To verify that LND-mito forms hydrogels instead of micelles, the critical micelle concentration (CMC) was determined using pyrene as a probe. The CMC value was determined to be as high as 0.63 mM (Figure 2D). LND-mito release from the hydrogel was subsequently measured in PBS at 37 °C With gradual disassembly of the hydrogel into molecular monomers, LND-mito was released sustainably, and the cumulative release reached 46.88% over 25 days (Figure 2E).

### 2.2. LND-Mito Actively Targets Mitochondria and Triggers Mitochondrial Functional Impairment

**Figure 3:**
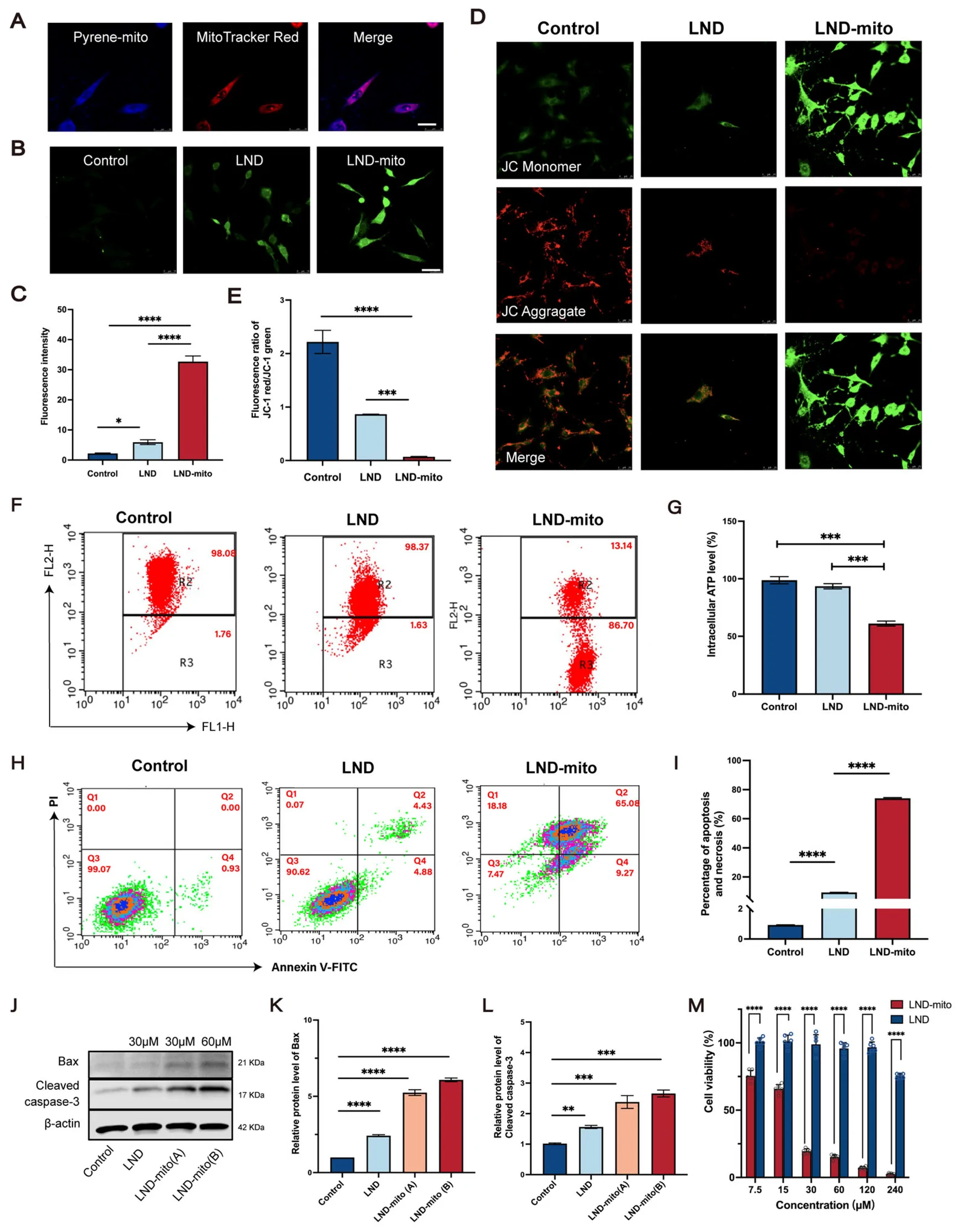
Mitochondria-targeting efficacy and mitochondrial dysfunction induced by LND-mito. (**A**) Co-localization of Pyrene-mito with mitochondria in GL261 cells. Scale bar = 5 μm. (**B**) Visualization of intracellular ROS fluorescence (DCFH-DA, green) and (**C**) its semiquantitative analysis in GL261 cells after 24 h treatment with 30 µM LND or LND-mito. (**D**) JC-1 staining for mitochondrial membrane potential in GL261 cells after 24 h exposure to 30 µM LND or LND-mito. (**E**) Semi-quantitative analysis of mitochondrial membrane potential based on the JC-1 red/green fluorescence ratio. (**F**) Flow cytometric analysis of mitochondrial membrane potential in GL261 cells treated with different formulations. (**G**) Relative ATP levels in GL261 cells after 24 h exposure to 30 µM LND or LND-mito. (**H**) Apoptosis profiles of GL261 cells. The x- and y-axes represent Annexin V-FITC and propidium iodide fluorescence signals, respectively. (**I**) Quantification of apoptotic and necrotic cell populations by flow cytometry. (**J**) Cleaved caspase-3 and Bax levels in GL261 cells after incubation with different concentrations of LND and LND-mito. (**K**,**L**) Quantitation of Bax and cleaved caspase-3 protein levels. (**M**) In vitro cytotoxicity of LND and LND-mito against GL261 cells at various concentrations. Data are means ± SDs (*n* = 3). Statistical significance was defined as * *p* < 0.05, ** *p* < 0.01, *** *p* < 0.001, **** *p* < 0.0001 by one-way ANOVA.

Mitochondria produce most cellular ATP via oxidative phosphorylation, regulate cell signaling and fate, and serve as the main reactive oxygen species (ROS) source. Excessive ROS production triggers oxidative stress and mitochondrial dysfunction, leading to cell death [34,35,36]. To validate the mitochondrial targeting ability of the designed peptide, we first synthesized Pyrene mito (Pyrene (CH_2_)_5_ CONH FFDRFKFDRFK NH_2_) by replacing LND with the blue fluorescent probe pyrene butyric acid (Figure S3). Confocal laser scanning microscopy (CLSM) showed that Pyrene-mito rapidly entered cells and exhibited excellent mitochondrial targeting (Figure 3A). Mitochondrial ROS levels were monitored using the DCFH DA fluorescent probe. As shown in Figure 3B,C, significantly higher ROS production was induced by LND-mito compared with free LND.

Mitochondrial membrane potential drives ATP synthesis and reflects mitochondrial function. LND affects this potential in association with ROS generation. JC-1 fluorescence was used to monitor mitochondrial membrane potential. In normal mitochondria, JC 1 accumulates and forms aggregates emitting red fluorescence. When mitochondria are damaged, JC 1 remains monomeric and generates green fluorescence. The red/green fluorescence ratio reflects mitochondrial membrane potential. As shown in Figure 3D,E, CLSM results revealed that LND-mito treatment markedly decreased red fluorescence while increasing green fluorescence, whereas intense red fluorescence was detected in both the control and LND groups. Consistently, flow cytometry revealed a markedly lower red/green ratio in LND-mito-treated cells (Figure 3F), indicating that LND-mito effectively reduced the mitochondrial membrane potential of GL261 cells.

Furthermore, cellular ATP levels, a direct indicator of mitochondrial energy metabolism, were measured. Decreased ATP levels signify impaired mitochondrial function. The LND-mito group displayed significantly lower ATP levels relative to the control and LND groups (Figure 3G), confirming that mitochondrially targeted LND more effectively disrupts mitochondrial function. Consequently, mitochondria-mediated intrinsic apoptosis was evaluated. Flow cytometry analysis revealed that LND-mito treatment induced significantly higher early and late apoptotic rates (65.08% and 9.27%, respectively) compared to free LND (4.43% and 4.88%, Figure 3H,I). Western blotting analysis was performed to elucidate the underlying apoptotic mechanism. As shown in Figure 3J–L, cleaved caspase 3 and Bax were markedly elevated by LND-mito versus control and LND groups, in a concentration-dependent manner. We selected GL261 cells to compare the cytotoxicity of LND and LND-mito. Cell inhibition increased in a concentration dependent manner, with IC_50_ values of 23.33 µM for LND-mito and 444.44 µM for LND, respectively (Figure 3M). We further evaluated the in vitro cytotoxicity against several glioblastoma cell lines and the human lung alveolar basal epithelial cell line A549 (Figure S4, Table S1). Consistent with above results, LND-mito exhibited approximately 20-fold higher cell killing activity than LND. The above results demonstrate that LND-mito can effectively target mitochondria, induce mitochondrial dysfunction and cell apoptosis, and ultimately lead to cell death.

### 2.3. LND-Mito Exerts Potent In Vivo Anti-Tumor Effects in Subcutaneous Glioblastoma Models

**Figure 4:**
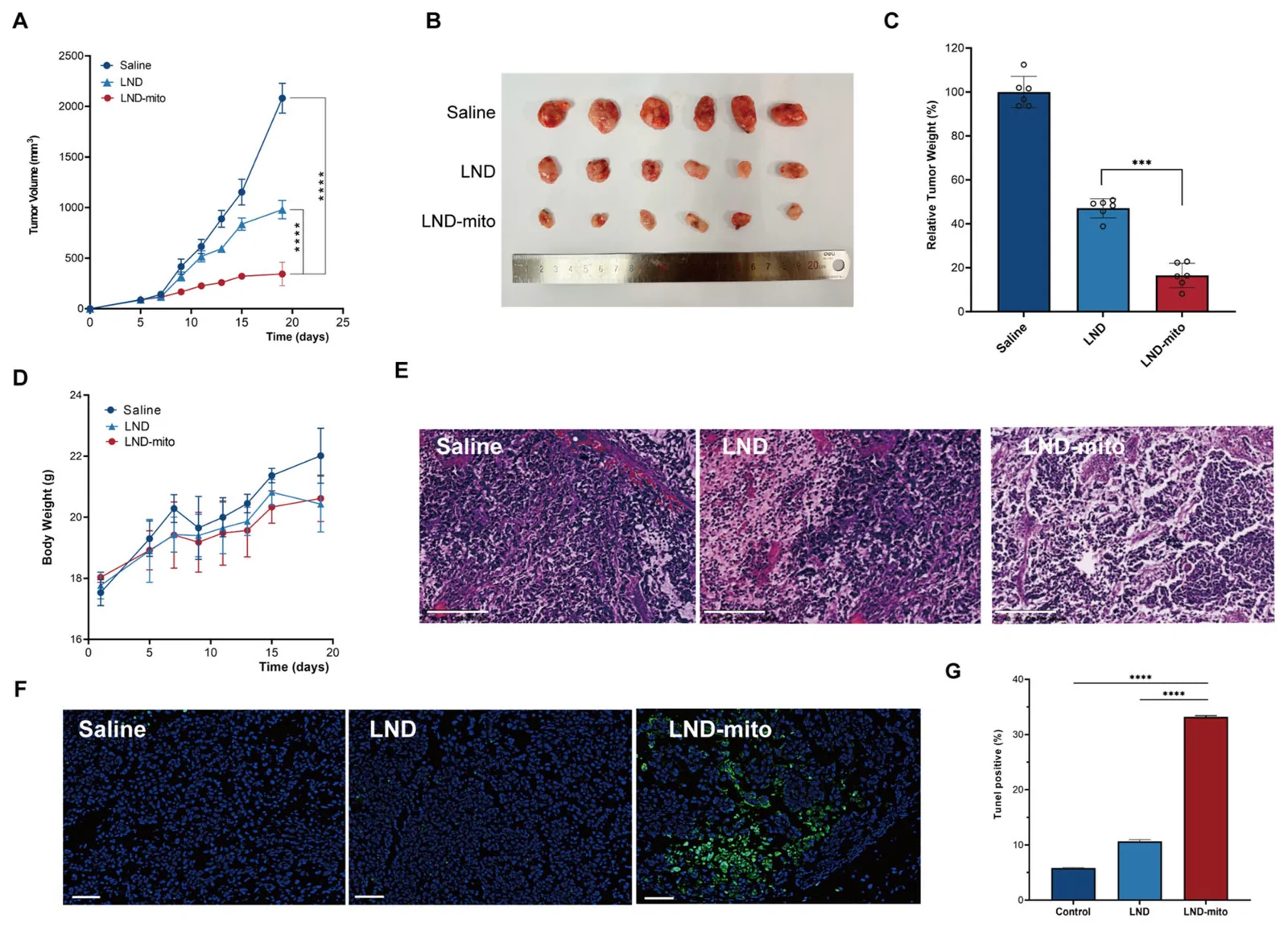
Antitumor efficacy of LND mito hydrogel in mice bearing subcutaneous GL261 glioma tumors. A subcutaneous GL261 glioma mouse model was established. Mice were intratumorally injected with 20 μL normal saline (control), 20 μL 250 mM LND, or 20 μL 250 mM LND mito, respectively, when tumor volumes reached 100–120 mm^3^. (**A**) Tumor growth curves in GL261-bearing mice after intratumoral injection (*n* = 6). (**B**) Excised tumor images on day 19. (**C**) Final tumor weights. (**D**) Body weight changes during treatment. Representative H&E staining (**E**) and TUNEL immunohistochemical staining images (**F**) of subcutaneous tumor sections. Scale bar = 100 μm. (**G**) Quantification of TUNEL positive apoptotic cells in tumor tissues after various treatments (*n* = 3). Data are means ± SDs. Statistical significance was defined as *** *p* < 0.001, **** *p* < 0.0001 by one way ANOVA.

The anti-tumor efficacy of LND-mito hydrogels was evaluated via intra-tumoral administration in mice bearing GL261 subcutaneous gliomas, with treatments initiated once tumors reached a volume of 100–120 mm^3^. As illustrated in Figure 4A, LND-mito hydrogels exhibited the most potent anti-tumor efficacy, with the ranking of tumor growth inhibition being LND-mito > LND > saline. Consistently, the mean tumor volume in LND-mito group was markedly lower than that in the saline and LND groups (Figure 4B,C). No obvious systemic adverse effects were observed throughout the treatment period, as confirmed by the absence of significant body weight fluctuations in mice (Figure 4D) and no noticeable histological abnormalities or pathological damage in all examined major organs (Figure S5). H&E staining revealed a significantly lower tumor cell density in the LND-mito group compared to the control group (Figure 4E). Furthermore, TUNEL immunohistochemistry was performed to detect apoptotic cells in tumor tissues, revealing a significantly increased apoptotic cell percentage in the LND-mito hydrogels group (Figure 4F,G). Collectively, these findings demonstrate that LND-mito hydrogels exerted significantly superior anti-tumor efficacy compared with free LND in the GL261 subcutaneous tumor model.

### 2.4. LND-Mito Exhibits Potent Postoperative In Vivo Anti-Tumor Effects in Orthotopic Glioblastoma Models

**Figure 5:**
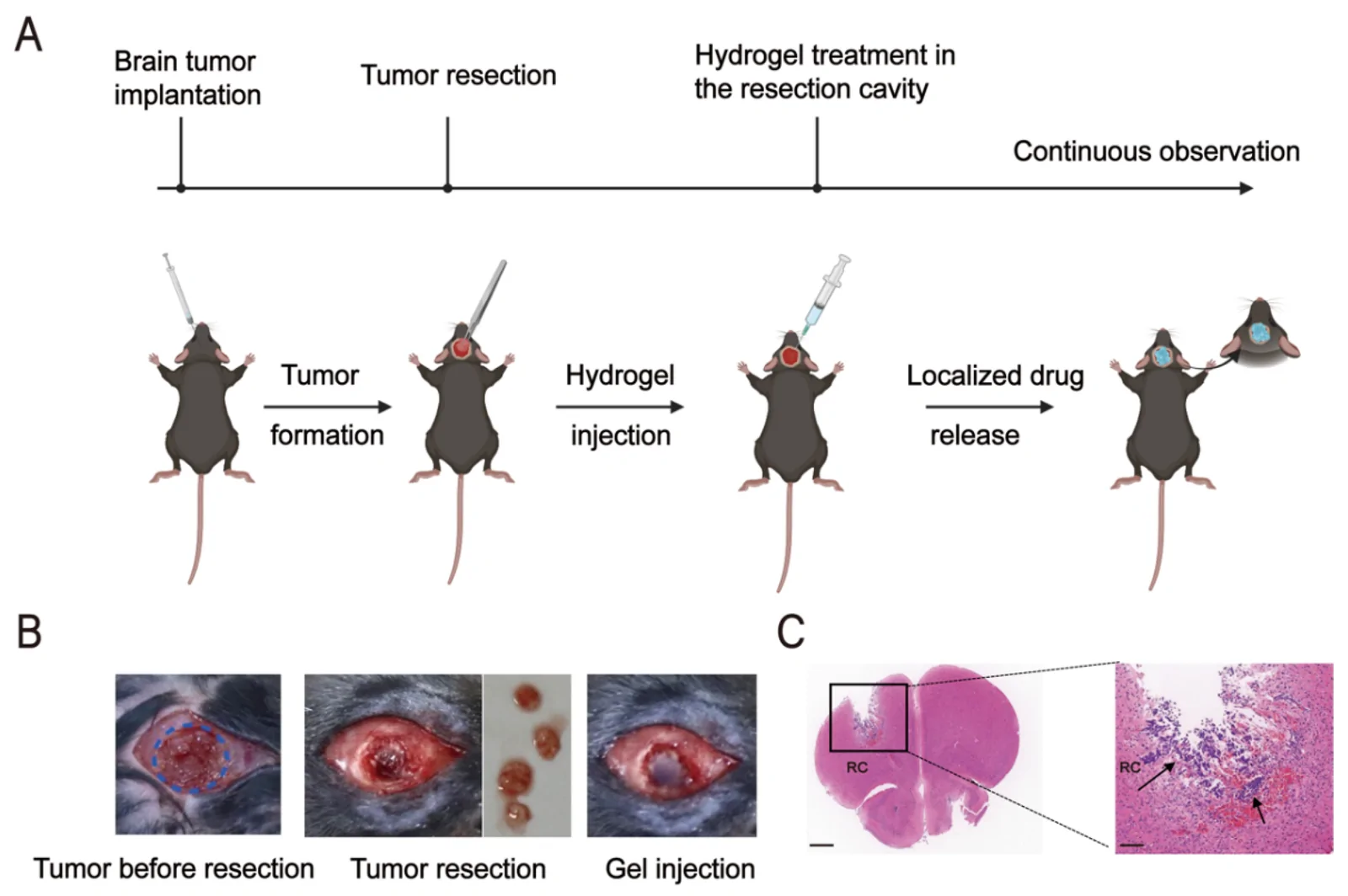
Postsurgical glioblastoma recurrence model establishment and sequential in situ hydrogel treatment. (**A**) Schematic depicting the construction of a postsurgical glioblastoma recurrence mouse model and the subsequent localized hydrogel therapy regimen following tumor resection. (**B**) Schematic workflow of surgical tumor resection and subsequent hydrogel implantation into the tumor resection cavity. (**C**) H&E-stained brain sections harvested immediately after total resection from GL261-bearing mice. RC denotes the resection cavity. Scale bars: 500 µm (left), 100 µm (right).

As surgical resection remains the standard treatment for glioblastoma, we established an in vivo model of incomplete glioma resection to evaluate the efficacy of LND-mito hydrogels as a postoperative adjuvant therapy. Briefly, orthotopic GL261-Luc glioma models were established in C57BL/6 mice. On day 12 after tumor inoculation, most of the tumor mass was surgically removed, with approximately 5% residual tissue retained to mimic minimal residual disease. After euthanasia, brain tissues were harvested for histology. H&E staining of brain sections clearly revealed the surgical resection cavity and residual tumor tissue (Figure 5).

**Figure 6:**
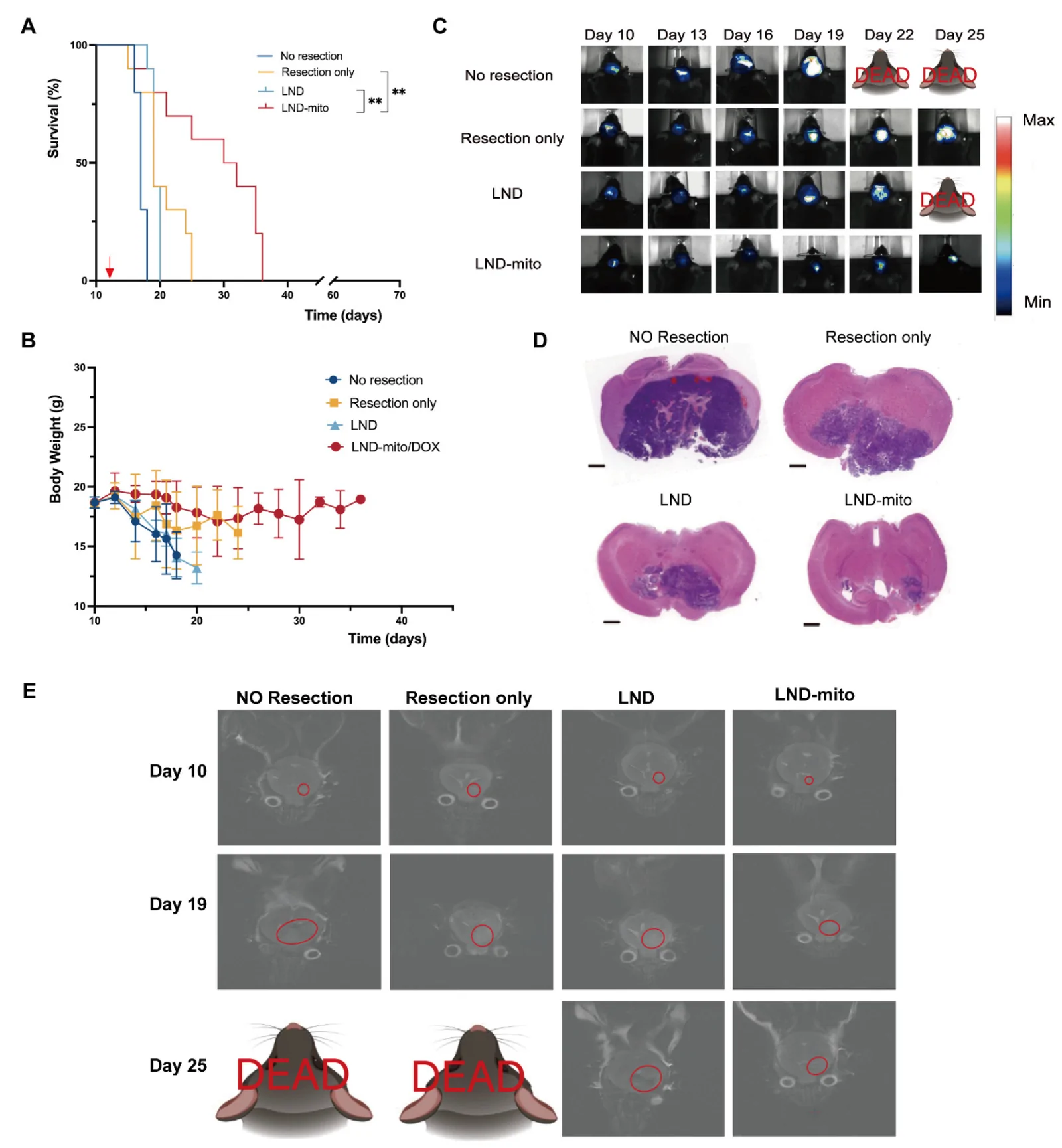
Inhibition of postsurgical brain glioblastoma recurrence by LND-mito hydrogels. (**A**) Survival analysis of mice bearing orthotopic GL261 glioblastoma following the indicated treatments (*n* = 10). (**B**) Body weight monitoring of mice subjected to different postsurgical treatments (*n* = 10). (**C**) Representative in vivo IVIS fluorescence images of postsurgical GL261 glioblastoma in response to designated treatments. (**D**) Representative magnetic resonance imaging (MRI) scans showing tumor resection and sequential in situ hydrogels treatment processes. (**E**) Typical H&E staining of mouse brain sections from each treatment group, depicting postsurgical glioma recurrence and proliferation. Scale bar = 500 μm. Data are as means ± SDs. Statistical significance was defined as ** *p* < 0.01.

Then, LND-mito hydrogel (500 mM/5 μL), LND (500 mM/5 μL), or saline was topically applied into the resection cavity. Tumor progression was monitored by MRI and IVIS, while body weight and survival rate were recorded. As shown in Figure 6A,B, tumors in the unresected group grew rapidly, accompanied by progressive body weight loss, and all mice succumbed to tumor burden within 17 days. The median survival was 19 days for both the saline and LND groups, whereas that of the LND-mito hydrogel group was markedly prolonged to 31 days, with one mouse remaining alive at day 40. These results demonstrated that local administration of LND-mito hydrogels into the tumor resection cavity can effectively complement surgical intervention, suppress tumor growth and improve survival. It is noteworthy that despite the significant prolongation of mouse survival conferred by hydrogel treatment, recurrent tumor growth was still detectable (Figure 6C–E). This observation highlights the necessity for further optimization of this therapeutic strategy. Future directions may include refining the design of the mito peptide sequence or engineering the hydrogel as a sustained-release delivery platform to co-deliver additional antitumor agents, thereby achieving synergistic anti-tumor effects and potentially eradicating residual disease completely.

### 2.5. Mechanistic Insights into LND-Mito Therapy by RNA Sequencing

**Figure 7:**
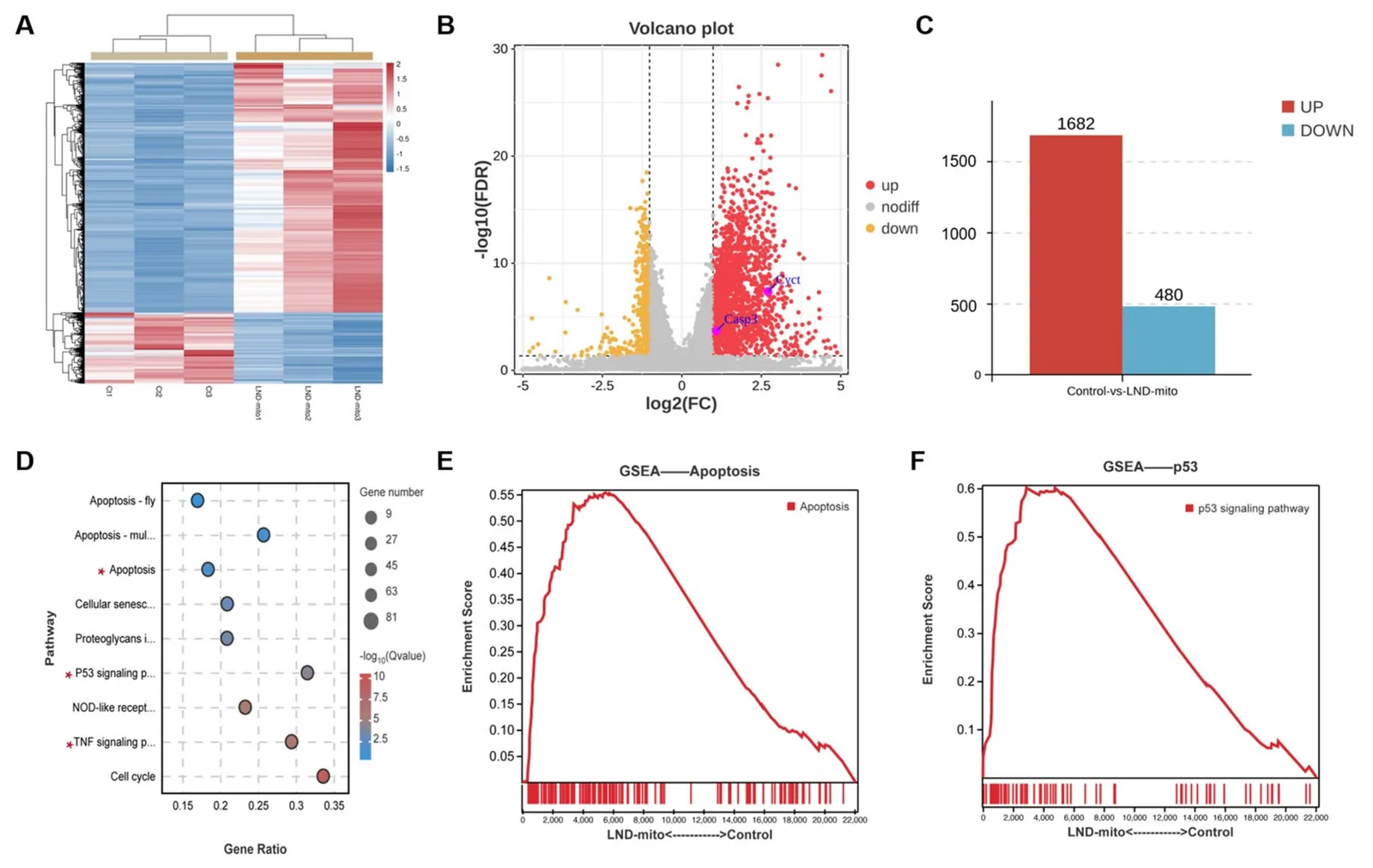
RNA sequencing analysis of glioblastoma mice brain tissues treated with LND-mito. (**A**) Hierarchical clustering of differentially expressed genes (DEGs) with normalized abundance between LND-mito and control groups (fold change > 1.5, *p* < 0.05). Rows = genes, columns = replicates (*n* = 3). (**B**) Volcano plot of DEGs between control and LND-mito. Dots with FDR ≤ 0.001 and ≥2-fold change: red = up, yellow = down. (**C**) Quantity of DEPs in different comparative groups. (**D**) Top 9 significantly enriched pathways of DEGs identified via KEGG analysis (* LND-mito treatment significantly upregulated genes associated with multiple apoptosis-related pathways). (**E**,**F**) GSEA enrichment maps showing that DEGs are mainly enriched in the apoptosis signaling pathway (**E**) and p53 signaling pathway (**F**).

To further explore the therapeutic mechanism of LND-mito against glioblastoma, transcriptome sequencing was implemented to profile overall gene expression alterations and identify differentially expressed genes (DEGs). Hierarchical clustering heatmap analysis showed that the mRNA expression profile of LND-mito–treated brain tissues was clearly distinct from that of the saline control group. As shown in Figure 7A, a total of 22,063 genes were detected in both the control and LND-mito groups. In the volcano plot (Figure 7B), the vertical axis represents the negative log10-transformed false discovery rate (FDR), with red and yellow dots indicating significantly upregulated and downregulated genes in LND-mito versus control, respectively. Overall, 1682 genes were upregulated and 480 downregulated (Figure 7C), demonstrating remarkably altered transcriptomic profiles upon LND-mito treatment. Bioinformatic analyses were performed to further characterize the biological functions of genes regulated by LND-mito (Figure 7D–F). KEGG pathway enrichment revealed that LND-mito treatment significantly upregulated genes associated with multiple apoptosis-related pathways, including apoptosis, the p53 signaling pathway, and the TNF signaling pathway. Consistently, KEGG-based gene set enrichment analysis GSEA further confirmed that apoptosis and the p53 signaling pathway were notably upregulated in LND-mito versus control.

Collectively, these transcriptomic profiles reveal the molecular mechanisms underlying the anti-glioma effects of LND-mito at the pathway level and provide robust evidence supporting its therapeutic potential against GBM by modulating key apoptotic signaling pathways.

### 2.6. LND-Mito Hydrogel Possesses Favorable Biocompatibility

Hemolysis assay was performed using rat red blood cell suspensions. The hemolytic rates of free LND and LND-mito were 2.78% and 3.33%, respectively, demonstrating favorable hemocompatibility (Figure S6). Subsequently, 10 µL of LND-mito hydrogel or LND solution was administered subcutaneously, and skin tissues around the injection site were harvested for histology. H&E staining showed no obvious inflammatory responses in skin tissues after LND-mito hydrogel administration (Figure S7). Taken together, these results confirm that LND-mito hydrogel exhibits excellent biocompatibility in mice, supporting its potential as a promising postoperative adjuvant for glioblastoma.

## 3. Discussion

In this work, we developed a mitochondria-targeted peptide–drug conjugate by covalently linking mitochondrial-penetrating peptides (mito) with the hexokinase II inhibitor lonidamine (LND). This amphiphilic LND-mito conjugate self-assembled into a carrier-free supramolecular hydrogel, offering local sustained chemotherapy to bridge the therapeutic gap between glioblastoma surgical resection and systemic treatment, inhibit residual tumor recurrence, and minimize systemic adverse reactions. By rationally engineering the peptide sequence to balance mitochondrial tropism and supramolecular gelation capacity, the LND-mito hydrogel exhibited favorable nanofiber self-assembly and tunable sustained release profiles. In vitro cellular assays confirmed that LND-mito exerted approximately 20-fold stronger cytotoxicity against glioma than free LND. Mechanistically, the conjugate selectively accumulated within tumor mitochondria, disrupted mitochondrial bioenergetics, and triggered robust apoptotic cell death. Consistent therapeutic benefits were validated in both subcutaneous and orthotopic postoperative glioma mouse models, where localized hydrogel administration markedly restrained tumor progression and distant recurrence. This peptide drug conjugate (PDC) platform features a unique “drug-as-carrier” design; the single molecular entity serves as both gel building block and therapeutic payload, and the system holds potential for co-loading additional chemotherapeutics such as doxorubicin to achieve synergistic anti-tumor effects.

As a rigid polyanhydride implant, Gliadel^®^ suffers from obvious drawbacks including early burst release, limited 3-week therapeutic window, poor cavity conformability, incomplete polymer degradation, narrow tissue diffusion range and non-selective cytotoxicity that easily induces drug resistance. In contrast, our injectable LND-mito precursor fits arbitrarily shaped resection cavities and forms intact hydrogel in situ. Relying on slow β-sheet nanofiber disassembly, it achieves burst-free sustained delivery over 25 days. Distinct from Gliadel^®^ whose copolymer leaves undegraded polymer residues and triggers long-term intracranial inflammation, the carrier-free LND-mito hydrogel is built on natural amino acid backbones that can be gradually degraded and eliminated via endogenous proteolysis and systemic metabolism, conferring intrinsically low immunogenicity and avoiding persistent foreign-body irritation. Dissociated LND-mito monomers diffuse widely in interstitial fluid and selectively target tumor mitochondria, killing residual infiltrative glioma while sparing normal neural tissue, which greatly reduces neurotoxicity. Moreover, the single-component design avoids multi-component batch instability seen in Gliadel^®^ and similar polymer implants.

Despite the promising findings above, this study has some inherent limitations. All in vivo experiments areconducted on mouse orthotopic glioma models, and species differences may hinder direct translation to human clinical practice. Current biocompatibility evaluation is limited to short-term histological observation. Long-term intracranial chronic inflammation, immune responses and the full in vivo metabolic degradation process of the peptide hydrogel have not been quantitatively assessed. Besdides, in vitro release was only monitored for 25 days. Due to compact internal β-sheet packing, much LND-mito is trapped within nanofiber assemblies, and the full degradation and in vivo clearance profile of the remaining hydrogel have not been quantified. Moreover, only monotherapy with LND-mito hydrogel is investigated in this work. Synergistic combination regimens and possible drug resistance arising from prolonged single-agent treatment remain to be explored in subsequent studies.

## 4. Conclusions

In summary, this study presents an innovative mitochondria-targeted supramolecular hydrogel that acts as both therapeutic agent and delivery carrier for localized postoperative glioblastoma treatment. By integrating tumor-specific mitochondrial enrichment, in-situ injectable gelation and long-term controlled release into one single peptide–drug conjugate molecule, this study establishes a straightforward and biosafe strategy for precise regional chemotherapy against invasive brain tumors.

## 5. Materials and Methods

### 5.1. Materials

Rink Amide-AM resin, LND, dichloromethane (DCM), N,N-dimethylformamide (DMF), N,N′-diisopropylcarbodiimide (DIC), and 1-hydroxybenzotriazole monohydrate were obtained from Aladdin Reagent Corporation (Shanghai, China). F-12K medium, fetal bovine serum (FBS), penicillin-streptomycin (P/S) and Dulbecco’s Modified Eagle Medium (DMEM) were purchased from Gibco (Grand Island, NY, USA). JC-1 mitochondrial membrane potential assay kits and Dichlorodihydrofluorescein diacetate (DCFH-DA) were purchased from MedChem Express (Monmouth Junction, NJ, USA). Phenylmethanesulfonyl fluoride (PMSF), CCK-8, MitoTracker Red CMXRos and ATP assay kits were sourced from Beyotime (Shanghai, China).

### 5.2. Cells and Animals

The GL261 cell line was purchased from Bohui Biotechnology Co., Ltd (Guangzhou, China). C57 BL/6 mice (female, 18–22 g, 6–8 weeks) were purchased from Shanghai Bikai Laboratory Animal Technology Co., Ltd (Shanghai, China). The mice were housed in a barrier environment with an ambient temperature of 24 °C. All animal experiments were performed in accordance with the experimental animal care and use guidelines of the Naval Medical University, and the experiments were approved by the Animal Ethics Committee of the Naval Medical University (approved on 3 October 2023).

### 5.3. Synthesis of LND-Mito

Fmoc-Lys-OH, HOBt, and DIC were placed in a constant-temperature oscillating shaker in a gas bath and activated at 35 °C for 5–10 min. They were then reacted with Rink Amide-AM resin without the Fmoc protective group at 60 °C in a constant-temperature shaking bed in a gas bath for 20 min. Then, the resin was cleaned three times with DMF, DCM, and DMF. The LND-(CH2)_5_-CONH-FFDRFKFDRFK-NH_2_ sequence was successively connected using the Fmoc-Lys-OH reaction. Next, Fmoc removal from the terminal residue was performed with 20% piperidine. Thereafter, a trifluoroacetic acid/phenol/water/triisopropylsilane mixture (volume ratio: 88/5/5/2; 20 mL) was added. The resin was transferred to a gas-bath thermostatic shaker at 35 °C for 3 h and then filtered out. Trifluoroacetic acid was blown out using argon, cold ether was added, shaken, and centrifuged, and the supernatant was discarded. This process was repeated three times. After ether evaporation and vacuum freeze-drying for 24 h, the crude polypeptide drug conjugate was collected and stored at −20 °C.

### 5.4. Preparation and Characterization of Hydrogels

A known amount of LND-mito was added to PBS (pH 7.4) and ultrasonicated for 2–3 min, and the hydrogel was obtained by stirring at room temperature. The polypeptide hydrogel was prepared at a concentration of 145 mM, and 10 µL of hydrogel was dropped on the carbon film. After negative staining, the microscopic morphology of the hydrogels was observed by TEM (FEI Talos F200X G2, accelerated electricity: 200 kV, Thermo Fisher Scientific, Hillsboro, OR, USA). The CD spectrum of the LND-mito formed in a 1-mm quartz cell was recorded in the 190–240 nm range with a JASCO J-1500 spectrometer (Tokyo, Japan). The CONTIN algorithm was used to evaluate the relative content of secondary structures for verification. An LND-mito solution (6 mM) was prepared and diluted stepwise to prepare a series of samples with a gradient concentration. Hydrophobic pyrene was used as a fluorescent probe to measure the critical micelle concentration (CMC) of the self-assembled polypeptides. The pyrene fluorescence spectra were obtained using a fluorescence spectrophotometer (Hitachi F-7000, Tokyo, Japan). The I_1_/I_3_ ratio of pyrene (370–373 nm/381–384 nm) was plotted against log_10_C to determine the CMC of polypeptides.

### 5.5. In Vitro Drug Release

The hydrogel was prepared in a 1 mL vial at a concentration of 145 mM. After aging for 6 h, PBS (400 µL, pH 7.4) was slowly added to the upper layer of the hydrogel. The sample was then placed in a thermostatic shaker at 37 °C and 100 rpm. At the specified time, all the PBS in the upper layer was retrieved. Subsequently, fresh PBS (400 µL) was added to the surface of the hydrogel. Cumulative drug release and release curve were calculated and plotted.

### 5.6. Verification of Mitochondrial Targeting

Pyrene-mito was synthesized by replacing the LND in the LND-mito structure with pyrene. GL261 cells were seeded in confocal plates (2 × 10^5^/well), incubated at 37 °C for 24 h, then treated with 4 µM pyrene-mito in DMEM for 4 h. Then, the sample was cleaned with PBS, and stained with the MitoTracker Red CMXRos (100 nM) probe at 37 °C for 30 min. After two cycles of rinsing with PBS, fresh PBS was added to keep the samples wet, and imaging was performed using CLSM (Leica Microsystems, Wetzlar, Germany).

### 5.7. Intracellular ROS Generation

Intracellular ROS production in GL261 cells treated with LND-mito was monitored using DCFH-DA. Intracellular ROS can oxidize non-fluorescent DCFH to produce fluorescent DCF. Cells were treated with 30 µM LND-mito or LND for 24 h, stained with 2 µM DCFH-DA for 20 min, and imaged by CLSM. Then the relative fluorescence intensity of the cells was determined using ImageJ software (version 1.8.0; Bethesda, MD, USA).

### 5.8. Changes in the Mitochondrial Membrane Potential

Mitochondrial membrane potential changes in LND-mito-treated GL261 cells were assessed using the JC-1 assay. In brief, the cells were first incubated with LND-mito (30 µM) for 24 h, then incubated with JC-1 (2 µM) in the dark for 30 min, washed with PBS, and analyzed by CLSM and flow cytometry. The histogram of the cells was processed using the FlowJo software v10.8.1 (BD Biosciences, Ashland, OR, USA), and fluorescence intensity per image was quantified with ImageJ, and the red/green ratio was calculated.

### 5.9. Determination of the Intracellular ATP Levels

In brief, GL261 cells were treated with LND-mito (30 µM), PBS, or LND (30 µM) for 24 h. Then, they were treated and washed according to kit instructions. Finally, a luminometer was used to measure the luminescence to quantify ATP.

### 5.10. Apoptosis

For apoptosis assay, cells in 6-well plates (5 × 10^5^/well) were treated with 30 µM LND-mito for 24 h. The cells were then collected, incubated with Annexin V-FITC and PI in the dark for 15 min, and analyzed by flow cytometry.

### 5.11. Western Blotting

Cells were lysed in RIPA buffer (Solarbio, Beijing, China) with 1 mM PMSF on ice. Protein was quantified by BCA. Samples (20 µg) were separated on 10% SDS-PAGE, transferred to PVDF membranes (Millipore, Burlington, MA, USA), blocked for 1 h at RT, and incubated with primary antibodies (β-actin 1:2000, cleaved caspase-3 1:1000, Bax 1:1000) overnight at 4 °C, then with secondary antibody (1:5000) for 1 h at RT. Blots were developed with ECL (Beyotime). The bands were quantified by analyzing the spectrum using ImageJ software.

### 5.12. Cell Viability Assay

In vitro cytotoxicity was assessed by CCK-8. GL261 cells (4 × 10^4^/well) in 96-well plates were incubated at 37 °C/5% CO_2_ for 24 h, treated with varying LND-mito or LND concentrations for another 24 h, then incubated with 10% CCK-8 for 1 h. Finally, absorbance at 450 nm was recorded.

### 5.13. Construction of Subcutaneous Glioma Model

Anti-tumor efficacy of LND-mito hydrogel was evaluated in C57 mice bearing subcutaneous GL261 tumors. When tumors reached 100–120 mm^3^, mice were randomized into five groups (*n* = 6) and received intratumoral injections: saline (20 µL, days 1 & 8), LND solution (250 mM/20 µL, days 1 & 8), or LND-mito hydrogel (250 mM/20 µL, single dose). Tumor size and body weight were monitored every 3 days The tumor volume was calculated by the formula: V = (major axis × (minor axis)^2^)/2. On day 19, mice were sacrificed, and the tumors were excised, photographed, and weighed. Then, the tumor as well as the major organs were excised and subjected to H&E staining. The stained sections were imaged by confocal microscopy.

### 5.14. Glioblastoma Tumor Resection Model and Local Treatment

Intracranial glioma was established in situ in C57 BL/6 mice. GL261-LUC cells (2 × 10^5^ in 5 µL of PBS) were stereotaxically injected into the brain (1.5 mm lateral, 0.6 mm anterior to bregma, 2 mm deep). To evaluate the effect of the LND-mito hydrogel on the anti-recurrence treatment of brain glioma, tumor resection was performed on day 12 following tumor inoculation. Glioma-bearing mice were randomly assigned to 5 groups (*n* = 10). After being anesthetized, the mice were fixed on a stereoscope, and a midline incision was made on the skin above the skull, with the seed cavity as the center. A skull drill with a diameter of 2 mm was used to perform craniotomy and complete tumor resection. Saline (5 µL), LND (500 mM/5 µL), LND-mito (500 mM/5 µL), were injected into the lesion site. The entire process was conducted on an animal insulation mat at an appropriate temperature.

The growth of the brain tumor was monitored by bioluminescence imaging using an IVIS spectral imaging system. The location and size of brain gliomas were also tracked through MRI using the horizontal-hole 7.0T Biospec Animal Imager (Bruker BioSpin, Ettlingen, Germany). The imaging conditions were TE: 52.56 ms, TR: 3 s, FOV: 30 mm × 30 mm, slice: 11, slice thickness: 0.8 mm, slice spacing: 0.2 mm, NEX: 2, ETL: 8, acquisition matrix: 320 × 320, and acquisition voxel: 0.093 mm × 0.093 mm. A T2-weighted image was obtained from the horizontal plane at predetermined time points. In addition, to examine tumor growth, mice were euthanized at various times, and brain specimens were collected, fixed, and stained with H&E.

### 5.15. Statistical Analysis

Data from at least three independent experiments are shown as mean ± SD. Statistical comparisons were made by Student’s *t*-test or one-way ANOVA using GraphPad Prism 9.0 (San Diego, CA, USA). Only data conforming to a normal distribution (*p* > 0.05) were subjected to parametric statistical analyses including unpaired Student’s *t*-test and one-way ANOVA. Statistical significance was set at *p* < 0.05.

## Data Availability

The original contributions presented in this study are included in the article. Further inquiries can be directed to the corresponding author.

## Supplementary Materials

The following supporting information can be downloaded at: https://www.mdpi.com/article/10.3390/gels12080682/s1, Figure S1: Structures of LND-mito compound used in the study; Figure S2: Structural characterizations of LND-mito; Figure S3: Structural characterizations of Pyrene-mito; Figure S4: Cytotoxicity of LND-mito in different cancer cell lines; Table S1: IC_50_ values of LND solution and LND-mito against various cancer cells (A549, U87, C6 and GL261) at 24 h; Figure S5: Representative H&E staining images of main organ tissues (heart, liver, spleen, lung, and kidney) from mice treated with saline, LND solution, and LND-mito hydrogel, respectively; Figure S6: Hemolytic ratios of LND and LND-mito (*n* = 3); Figure S7: Representative images of H&E staining images of skin tissues of C57BL/6 mice at injection sites of saline (I), LND (II), and LND-mito hydrogel (III).
