# Supplementary material for: Mitochondria-Targeted Lonidamine Hydrogels for Postsurgical Elimination of Glioblastoma
Source: Gels. 2026 Aug 3;12(8):682. doi: 10.3390/gels12080682 (PMC13512554; doi:10.3390/gels12080682)
Supplement: Supplementary file 1 [file gels-12-00682-s001.zip › gels-4407996-supplementary.pdf]

Supporting Information

Mitochondria-Targeted Lonidamine Hydrogels for Postsurgical Elimination of Glioblastoma

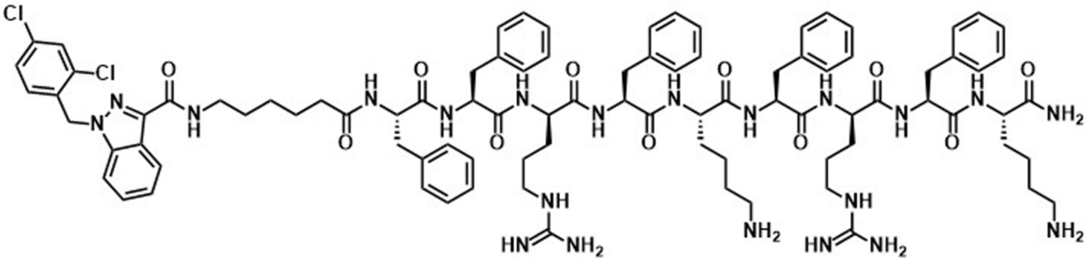

Figure S1. Structures of LND-mito compound used in the study.

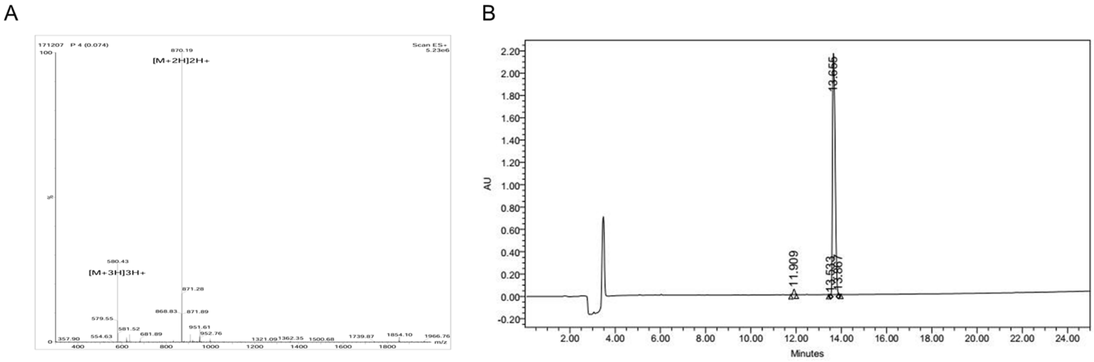

Figure S2. Structural characterizations of LND-mito. (A) TOF-MS: calc. M_W=1738.38, obsvd. $[M+2H]^{2H+}$ 870.19, $[M+3H]^{3H+}$ 580.43. (B) HPLC elution curve. Detailed HPLC analysis conditions were as follows: column temperature, 40 °C; C18 column (YMC, 5 μm, 4.6×250 mm); mobile phase A, 0.1% trifluoroacetic acid in ultrapure water; mobile phase B, 0.1% trifluoroacetic acid in acetonitrile; flow rate, 1 mL/min; detection wavelength, 220 nm.

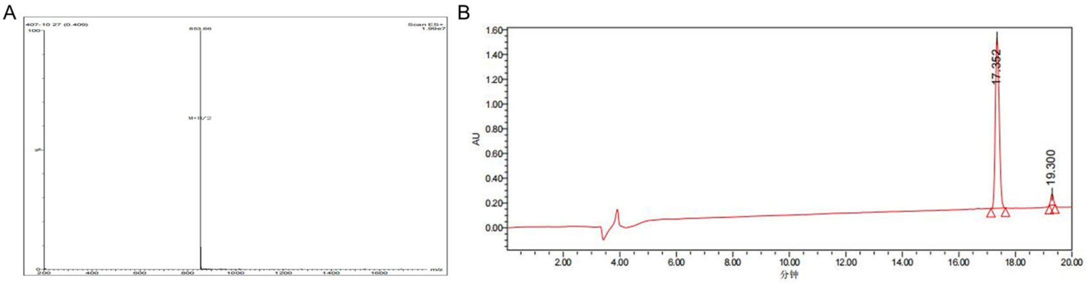

Figure S3. Structural characterizations of Pyrene-mito. (A) TOF-MS: calc. M_W=1706.38, obsvd. $[M+2H]^{2H+}$ 853.66. (B) HPLC elution curve. Detailed HPLC analysis conditions were as follows: column temperature, 40 °C; C18 column (YMC, 5 μm, 4.6×250 mm); mobile phase A, 0.1%

trifluoroacetic acid in ultrapure water; mobile phase B, 0.1% trifluoroacetic acid in acetonitrile; flow rate, 1 mL/min; detection wavelength, 220 nm.

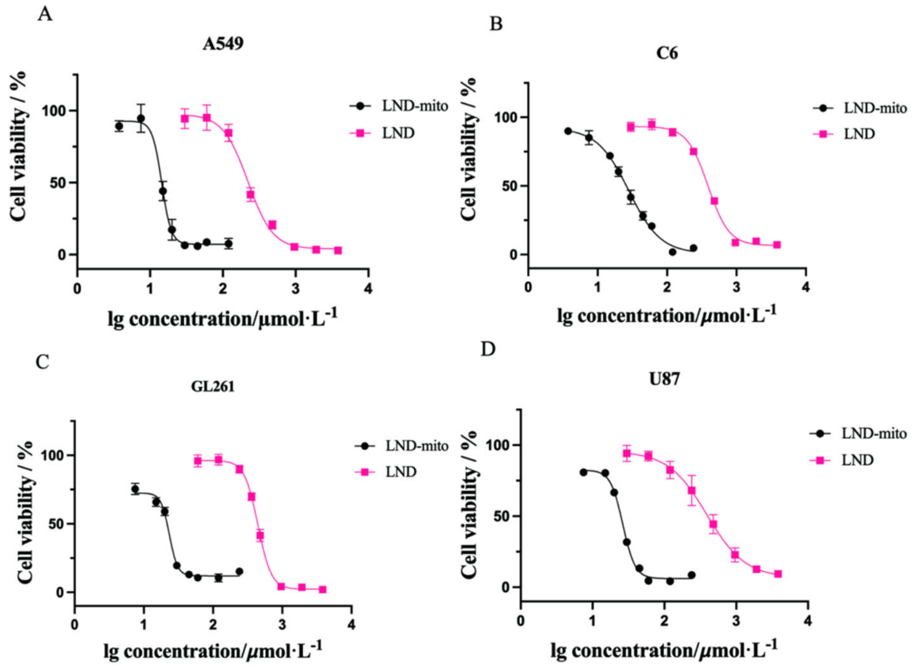

Figure S4. Cytotoxicity of LND-mito in different cancer cell lines. (A) A549; (B) C6; (C) GL261; (D) U87. Data represent mean ± SD from three independent experiments.

Table S1. IC_{50} values of LND solution and LND-mito against various cancer cells (A549, U87, C6 and GL261) at 24 h.

IC_{50}/μM	A549	U87	C6	GL261
LND	221.4	389.60	389.20	444.40
LND-mito	14.45	26.38	28.11	23.33

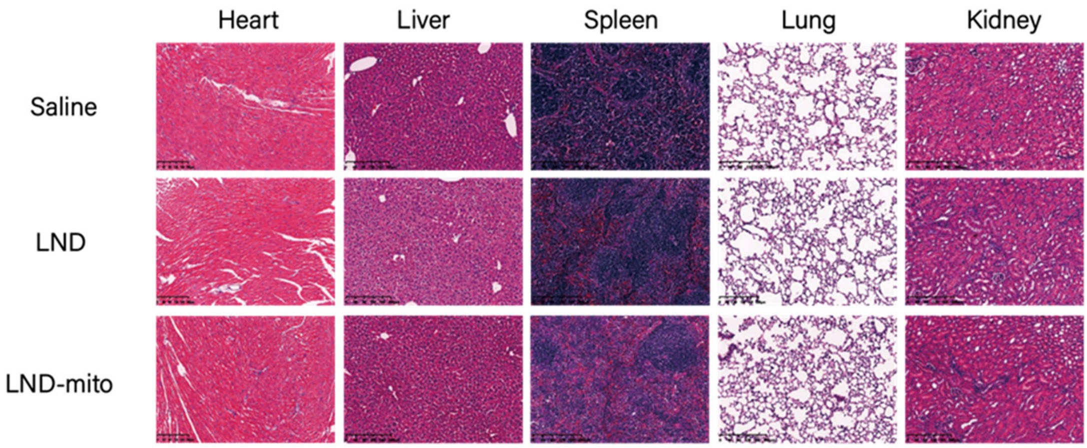

Figure S5. Representative H&E staining images of main organ tissues (heart, liver, spleen, lung, and kidney) from mice treated with saline, LND solution, and LND-mito hydrogel, respectively. Scale bar=200 μm.

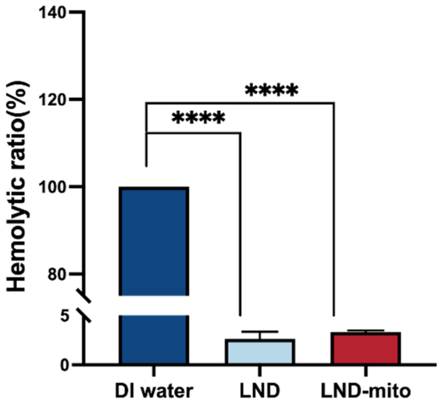

Figure S6. Hemolytic ratios of LND and LND-mito (n=3). Data are presented as means ± SDs. $\ast\ast\ast p < 0.0001$ by one-way ANOVA.

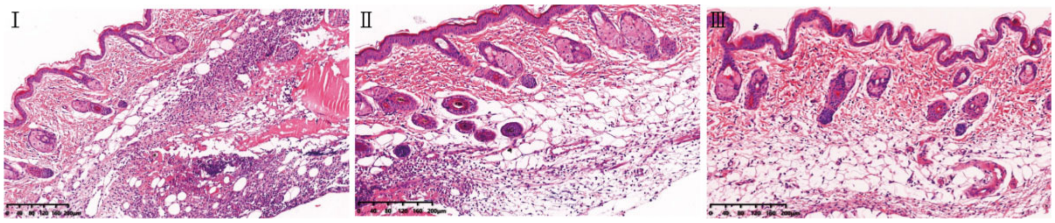

Figure S7. Representative images of H&E staining images of skin tissues of C57BL/6 mice at injection sites of saline (I), LND (II), and LND-mito hydrogel (III). Bar=200 μm.
